# The vitamin D receptor gene ApaI polymorphism is associated with increased risk of renal cell carcinoma in Chinese population

**DOI:** 10.1038/srep25987

**Published:** 2016-05-13

**Authors:** Chunming Yang, Jia LI, Yan Li, Di Wu, Chengguang Sui, Youhong Jiang, Fandong Meng

**Affiliations:** 1 Department of Urology, the First Hospital of China Medical University, Shenyang 110001, China; 2 Blood Collecting Centers, the First Hospital of China Medical University, Shenyang 110001, China; 3 Molecular Oncology Department of Cancer Research Institution, the First Hospital of China Medical University, Shenyang 110001, China

## Abstract

Molecular epidemiologic studies previously reported that 1,25-dihydroxy vitamin D_3_ (1,25(OH)_2_ D_3_) appears to influence cancer risk. It exerts its activity through the intracellular vitamin D receptor (VDR), which regulates the transcription of genes. This study aimed to investigate the genetic association of *VDR* polymorphisms with renal cell carcinoma (RCC) risk in the Chinese population. The genotypes of five *VDR* polymorphisms (TaqI, BsmI, Cdx-2, ApaI, and FokI) were studied using polymerase chain reaction in 302 RCC patients and 302 healthy controls. ApaI variant AA and AC genotypes were found to be associated with a significantly increased risk of RCC compared with the CC genotype (OR = 2.60, 95% CI = 1.39–4.85 for AA vs. CC, and OR = 1.52, 95% CI = 1.08–2.13 for AC vs. CC). The AA genotype was also associated with a higher Fuhrman grade (OR = 2.87, 95% CI = 1.15–7.16 for AA vs. CC). No significant difference was found between the other four *VDR* polymorphisms and RCC risk. Our study suggests that *VDR* ApaI genotypes may be involved in the increased risk and progression of RCC in the Chinese Han population.

Kidney cancer is the 13th most common malignancy worldwide, with around 270,000 cases diagnosed annually, and 116,000 people dying each year from the disease^1^. Approximately 90% of all kidney cancers are renal cell carcinomas (RCC), which are the most common type of urological cancer with a high prevalence in older men (>70 years of age)^2^,^3^. The causes of RCC are not completely known, although its development is associated with several risk factors, including obesity, smoking, hypertension, ethnicity, age, and family history^4^.

Epidemiological and laboratory investigations have shown that vitamin D deficiency is associated with several diseases, including autoimmune diseases, tuberculosis and cancer. Vitamin D maintains calcium homeostasis and regulates the growth and differentiation of various malignant tumors. Its activity is mediated through the binding of its active form, 1,25-dihydroxyvitamin D3 (1,25(OH)_2_ D_3_), to the vitamin D receptor (VDR), causing a conformational change and leading to dimerization with the retinoid X receptor. The dimeric complex then interacts with vitamin D response elements in target genes involved in cell division, cell adhesion, and function, resulting in changes in gene activity^5^. At the cellular level, the stimulation of VDR and retinoid receptors can cause the inhibition of cell proliferation and angiogenesis, as well as the induction of differentiation and apoptosis^6^. Because vitamin D activity is mediated by the VDR, the analysis of *VDR* genetic variation may elucidate the role of vitamin D in RCC etiology.

*VDR* is located on the long arm of chromosome 12q12-q14, and several single nucleotide polymorphisms (SNPs) within the gene have been identified that may influence cancer risk. Each polymorphism is named according to the restriction site that was initially used to identify it. The FokI (rs2228570) polymorphism is located at the first potential start site^7^, and alters an ACG codon that results in the generation of an additional start codon^8^. BsmI (rs1544410), ApaI (rs7975232), and TaqI (rs731236) polymorphisms are located in the 3′ untranslated region (UTR), and are all involved in regulating the stability of *VDR* mRNA^9^. Five well-known SNPs of human *VDR*, FokI (C/T), BsmI (A/G), ApaI (A/C), TaqI (T/C), and Cdx2 (A/G), were previously extensively studied for their association with cancer risk^8^,^10^. However, there is still a scarcity of data regarding the association of *VDR* genotype with RCC patients, and the results has not been consistent^11^,^12^,^13^.

Therefore, to determine whether variation in vitamin D receptor modify RCC risk, we conducted a case–control study to evaluate the association between *VDR* variants and RCC risk in the Chinese Han population.

## Results

Table 1 shows the general characteristics of cases and controls that were included in the present study. The mean age of cases was 54.76 years (standard deviation (SD) 10.96; range, 27–80 years), and that of controls was 54.17 years (SD 12.35; range, 24–87 years). No significant difference was observed between RCC patients and controls regarding age or sex. However, significantly more smokers (*P* = 0.006) and hypertensive patients (*P* = 0.029) were observed in the RCC group compared with the controls (Supplementary Information). Of the 302 RCC patients, 229 (75.83%) had RCC stage I–II, while 241 (79.80%) had RCC grade stage I–II.

### Association of ***VDR*** polymorphisms with RCC risk

Genotype and allele distributions of *VDR* polymorphisms in the cases and controls are shown in Table 2. In our study population, all genotype distributions in the control subjects were in agreement with HWE (Hardy-Weinberg equilibrium) (*P* = 0.82, *P* = 0.26, *P* = 0.69, *P* = 0.08, and *P* = 0.33 for TaqI, BsmI, Cdx-2, ApaI, and FokI, respectively). Multivariate logistic regression analyses showed that patients with AA and AC genotypes of the ApaI polymorphism had a significantly increased RCC risk compared with those with the CC genotype (adjusted OR = 2.60, 95% CI = 1.39–4.85 for AA vs. CC; OR = 1.52, 95% CI = 1.08–2.13 for AC vs. CC). The ApaI polymorphism A allele was also associated with an increased risk of RCC between cases and controls (*P* = 0.001). However, no significant association was found between variant genotypes of the other four *VDR* polymorphisms and risk of RCC (*P* > 0.05).

### ***VDR*** polymorphisms and RCC clinicopathologic characteristics

Table 3 shows the distribution of cases according to available RCC histopathological characteristics. We compared the various risk factors with stage and grade of RCC. The AA genotype of the ApaI polymorphism was found to be associated with a higher Fuhrman grade (OR = 2.87, 95% CI = 1.15–7.16 for AA vs. CC), but not with the AJCC (American Joint Committee on Cancer) stages of RCC (*P* = 0.766). There were no significant associations between the other four *VDR* polymorphisms and AJCC stages or Fuhrman grades in all patients.

## Discussion

In this study, we observed a significant association between the *VDR* ApaI variant and RCC risk in the Chinese Han population. Patients with AA and CA genotypes of the ApaI polymorphism were shown to have a 2.66- and 1.47-fold increased RCC risk, respectively, compared with those with the CC genotype. We also found that the ApaI polymorphism AA genotype was associated with a higher Fuhrman grade of RCC. However, none of the other four polymorphisms (TaqI, BsmI, Cdx-2, and FokI) were associated with the incidence of RCC. These results provide evidence for a potential role of inherited *VDR* variants in the development of RCC.

A few previous studies have comprehensively analyzed the genetic variation in *VDR* in relation to RCC. For example, a recent case–control study of 135 RCC patients and 150 controls in a Japanese population found that participants with the ApaI site AA genotype had a significant increase in RCC risk and a poor prognosis^11^. These data are consistent with those of our current report. However, because the ApaI SNP has only been analyzed in small studies typically limited to only one racial group, additional studies on larger population sizes are warranted to confirm these data. This SNP is located in intron 8 near the 3′ UTR of the VDR gene, and does not alter the amino acid sequence of the encoded protein; however, it may influence gene expression through the regulation of mRNA stability^15^. Recent genetic studies have shown that synonymous mutations may play an important role in disease etiology; they can affect protein expression and function by altering mRNA stability^16^,^17^.

FokI is the *VDR* SNP that has been most analyzed because of its functional significance. The FokI C allele encodes a VDR protein lacking three amino acids compared with that encoded by the T allele, which results in enhanced VDR transcription activity. In an earlier study conducted in Central and Eastern Europe, the prevalence of three common *VDR* SNPs (BsmI, FokI, and TaqI) did not differ according to tumor stage or grade^12^. These data are consistent with our own. In our study, RCC risk was not associated with the TaqI , BsmI and FokI polymorphisms. However, a recent study by Arjumand *et al.*, which analyzed associations of *VDR* SNP genotypes with RCC progression in terms of AJCC stage and nuclear grade, reported that FokI and BsmI were significantly associated with increased RCC risk among the North Indian population^13^. These discrepancies may reflect the limited number of cases in some studies, or the analysis of different ethnic groups. Indeed, it has been demonstrated that allelic distribution varies among different ethnic groups, which may lead to controversial results among different populations^18^,^19^,^20^. To our knowledge, this is the first study to examine Cdx-2 polymorphism and RCC risk. Cdx-2 leads to decreased transcriptional activity of the *VDR* promoter^21^. However, we found no association of Cdx-2 with RCC risk in our study.

Like other complex diseases, RCC appears to be caused by both genetic and environmental factors, such as cigarette smoking, obesity, and hypertension^22^,^23^. The sample size in our current study was relatively small, so it might not provide sufficient statistical power to assess the association between *VDR* SNPs and RCC risk. We didn’t perform further subgroup stratification analysis by smoking or obesity because of the insufficient of our data. Moreover, our study population was only of Han Chinese; thus, more comprehensive studies involving larger sample sizes of ethnically diverse populations are warranted to validate these findings.

In conclusion, this study indicates that the ApaI polymorphism of *VDR* is associated with RCC risk in the Chinese Han population. It also suggests that the ApaI polymorphism is a potential determinant of prognosis in terms of Fuhrman grade in RCC patients. Additional studies are needed to fully elucidate the role of *VDR* genetic variation in RCC risk.

## Methods

### Study population

A total of 302 RCC Chinese Han patients and 302 healthy controls were recruited between November 2009 and April 2012 at the First Hospital of China Medical University (Shenyang, China). All patients were recruited after a radiologic and histologic diagnosis of RCC. Detailed information about age, sex and clinical characteristics were obtained for all participants (see Table 1). The histologic subtype distribution was as follows: 241 clear cell RCC, 14 papillary RCC, 20 chromophobe RCC, 22 multilocular cystic RCC, and five unclassified RCC. Tumor stages and grades were determined according to the tumor–node–metastasis classification of the American Joint Committee on Cancer (AJCC) and the Fuhrman scale. Controls were randomly selected from individuals undergoing a health examination at the First Hospital of China Medical University during the same time period. Controls were cancer-free and were age- and sex-matched to cases. Informed consent was obtained from all individual participants included in the study. The study was approved by the Ethics Committee of the First Hospital of China Medical University, and carried out in accordance with the Declaration of Helsinki.

### DNA extraction and genotyping

Genomic DNA was extracted from 200 μL of whole blood samples using the QIAamp DNA Micro Kit (Qiagen, Hilden, Germany) according to the manufacturer’s instructions. Extracted DNA was dissolved to a concentration of 30 ng/μL and was stored at −80 °C. High-resolution melting methods using the LightCycler® 480 system (Roche, Mannheim, Germany) were used for SNP genotyping according to a previously reported protocol^14^. Primer sequences used for amplification are listed in Table 4. For quality control purposes, 20% of cases and controls were verified by direct sequencing to confirm SNP genotypes, resulting in a concordance rate of 100%. We confirmed our genotyping results by direct sequencing using an ABI3730 sequencer (ABI, America).

### Statistical analysis

A goodness-of-fit chi-squared (χ^2^) test was used to evaluate the Hardy–Weinberg equilibrium (HWE) of the gene frequencies of the healthy individuals. Differences in frequency distributions of epidemiological factors between RCC cases and controls were tested using the Student’s *t*-test for continuous variables and the χ^2^-test for categorical variables. To avoid spurious associations due to a small number of homozygous individuals, we used the dominant model for TaqI and BsmI to evaluate associations between the genotypes and RCC risk. The associations between *VDR* polymorphisms and RCC risk were estimated by calculating odds ratios (ORs) and 95% confidence intervals (CIs). from unconditional logistic regression analysis, adjusting for age and sex. The ORs and 95%CI were calculated by multivariate logistic regression analyses, adjusted for the effect of covariates like sex, age, smoking and hypertension status. All statistical tests were two-tailed with *P*-value < 0.05 considered as statistically significant. All statistical analyses were performed using the SPSS statistical package, version 19.0. (SPSS Inc., Chicago, IL).

## Additional Information

**How to cite this article**: Yang, C. *et al.* The vitamin D receptor gene ApaI polymorphism is associated with increased risk of renal cell carcinoma in Chinese population. *Sci. Rep.* **6**, 25987; doi: 10.1038/srep25987 (2016).

## Supplementary Material

Supplementary Information

## Figures and Tables

**Table 1:** Clinicopathologic characteristics of the renal cell carcinoma cases and controls.

Parameter	Cases, n (%)	Control, n (%)	P
Age (years)			
Mean ± SD	54.76 ± 10.96	54.17 ± 12.35	0.530
Range	27–80	24–87	
Sex			
Male	202 (66.89%)	195 (64.57%)	0.548
Female	100 (33.11%)	107 (35.43%)	
Hypertension			
No	217 (71.86%)	240 (79.47%)	0.029
Yes	85 (28.14%)	62 (20.53%)	
Smoking			
Never	204 (67.55%)	234 (77.48%)	0.006
Ever	98 (32.45%)	68 (22.52%)	
Stage			
T1	186 (61.59%)		
T2	43 (14.24%)		
T3	36 (11.92%)		
T4	37 (12.25%)		
Grade			
I	83 (27.48%)		
II	158 (52.32%)		
III	53 (17.55%)		
IV	8 (2.65%)		
Histopathologic subtype			
Clear cell renal cell carcinoma	241 (79.80%)		
Papillary renal cell carcinoma	14 (4.64%)		
Chromophobe renal cell carcinoma	20 (6.62%)		
Multilocular cystic renal cell carcinoma	22 (7.28%)		
Unclassified renal cell carcinoma	5 (1.66%)		

**Table 2:** Genotype and allele frequencies of the *VDR* polymorphisms in RCC cases and controls.

Genotype	Cases, n (%)	Controls, n (%)	Crude OR (95% CI)	*P* value	Adjusted OR (95% CI)*	*P* value*
TaqI (rs731236)						
TT	261 (86.42%)	272 (90.07%)	1.00 (reference)		1.00 (reference)	
CT + CC	41 (13.58%)	30 (9.93%)	1.42 (0.86–2.35)	0.166	1.43 (0.86–2.36)	0.169
T allele	563 (93.21%)	574 (95.03%)	1.00 (reference)			
C allele	41 (6.79%)	30 (4.97%)	1.39 (0.86–2.26)	0.180	1.41 (0.85–2.35)	0.179
BsmI (rs1544410)						
GG	255 (84.44%)	265 (87.75%)	1.00 (reference)		1.00 (reference)	
AG + AA	47 (15.56%)	37 (12.25%)	1.32 (0.83–2.10)	0.241	1.32 (0.83–2.11)	0.242
G allele	557 (92.22%)	567 (93.87%)	1.00 (reference)			
A allele	47 (7.78%)	37 (6.13%)	1.29 (0.83–2.02)	0.259	1.32 (0.83–2.10)	0.244
Cdx–2 (rs11568820)						
AA	100 (33.11%)	98 (32.45%)	1.00 (reference)		1.00 (reference)	
AG	153 (50.66%)	151 (50.00%)	0.99 (0.69–1.42)	0.969	0.96 (0.67–1.38)	0.832
GG	49 (16.23%)	53 (17.55%)	0.91 (0.56–1.46)	0.686	0.90 (0.56–1.46)	0.678
AG + GG	202 (66.89%)	204 (67.55%)	0.97 (0.69–1.36)	0.862	0.97 (0.69–1.36)	0.853
A allele	353 (58.44%)	347 (57.45%)	1.00 (reference)			
G allele	251 (41.56%)	257 (42.55%)	0.96 (0.76–1.21)	0.727	0.94 (0.72–1.22)	0.632
ApaI (rs7975232)						
CC	114 (37.75%)	149 (49.34%)	1.00 (reference)		1.00 (reference)	
AC	153 (50.66%)	135 (44.70%)	**1.48 (1.06**–**2.07)**	**0.022**	**1.52 (1.08**–**2.13)**	**0.016**
AA	35 (11.59%)	18 (5.96%)	**2.54 (1.37**–**4.72)**	**0.003**	**2.60 (1.39**–**4.85)**	**0.003**
AC + AA	188 (62.25%)	153 (50.66%)	**1.61 (1.16**–**2.22)**	**0.004**	**1.60 (1.16**–**2.22)**	**0.004**
C allele	381 (63.08%)	433 (71.69%)	1.00 (reference)			
A allele	223 (36.92%)	171 (28.31%)	**1.49 (1.17**–**1.90)**	**0.001**	**1.75 (1.31**–**2.32)**	**<0.001**
FokI (rs2228570)						
CC	70 (23.18%)	79 (26.16%)	1.00 (reference)			
CT	171 (56.62%)	159 (52.65%)	1.21 (0.82–1.79)	0.327	1.15 (0.78–1.71)	0.476
TT	61 (20.20%)	64 (21.19%)	1.08 (0.67–1.73)	0.764	1.05 (0.65–1.70)	0.835
CT + TT	232 (76.82%)	223 (73.84%)	1.17 (0.81–1.70)	0.396	1.16 (0.80–1.68)	0.444
C allele	311 (51.49%)	317 (52.48%)	1.00 (reference)			
T allele	293 (48.51%)	287 (47.52%)	1.04 (0.83–1.30)	0.730	1.05 (0.80–1.37)	0.746

^*^Adjusted for age, gender, smoking and hypertension in multivariate unconditional logistic regression model. The P values < 0.05 are indicated in bold.

**Table 3:** Association between *VDR* polymorphisms and grade and stage of RCC in all patients.

Genotype	Grade grouping, n (%)		Adjusted OR (95% CI)*	*P* value*	Stage grouping, n (%)		Adjusted OR (95% CI)*	*P* value*
	III + IV	I + II			III + IV	I + II		
TaqI (rs731236)								
TT	54 (88.52%)	207 (85.89%)	1.00 (reference)		59 (80.82%)	202 (88.21%)	1.00 (reference)	
CT + CC	7 (11.48%)	34 (14.11%)	1.02 (0.41–2.55)	0.962	14 (19.18%)	27 (11.79%)	1.79 (0.86–3.69)	0.117
BsmI (rs1544410)								
GG	49 (80.33%)	206 (85.48%)	1.00 (reference)		59 (80.82%)	196 (85.59%)	1.00 (reference)	
AG + AA	12 (19.67%)	35 (14.52%)	1.63 (0.75–3.53)	0.215	14 (19.18%)	33 (14.41%)	1.41 (0.70–2.84)	0.338
Cdx–2 (rs11568820)								
AA	26 (42.62%)	74 (30.71%)	1.00 (reference)		24 (32.88%)	76 (33.19%)	1.00 (reference)	
AG	25 (40.98%)	128 (53.11%)	0.55 (0.29–1.05)	0.071	37 (50.68%)	116 (50.65%)	0.98 (0.54–1.79)	0.959
GG	10 (16.40%)	39 (16.18%)	0.63 (0.27–1.48)	0.287	12 (16.44%)	37 (16.16%)	1.06 (0.47–2.37)	0.896
AG + GG	35 (57.38%)	167 (69.29%)	0.60 (0.33–1.09)	0.096	49 (67.12%)	153 (66.81%)	1.01 (0.57–1.76)	0.992
ApaI (rs7975232)								
CC	18 (29.51%)	96 (39.83%)	1.00 (reference)		30 (41.10%)	84 (36.68%)	1.00 (reference)	
CA	31 (50.82%)	122 (50.62%)	1.40 (0.72–2.76)	0.325	33 (45.20%)	120 (52.40%)	0.83 (0.47–1.49)	0.541
AA	12 (19.67%)	23 (9.55%)	**2.87 (1.15**–**7.16)**	**0.023**	10 (13.70%)	25 (10.92%)	1.14 (0.48–2.68)	0.766
CA + AA	43 (70.49%)	145 (60.17%)	1.55 (0.83–2.91)	0.170	43 (58.90%)	145 (63.32%)	0.86 (0.50–1.49)	0.863
FokI (rs2228570)								
CC	13 (21.31%)	57 (23.65%)	1.00 (reference)		17 (23.29%)	53 (23.14%)	1.00 (reference)	
CT	31 (50.82%)	140 (58.09%)	0.87 (0.41–1.85)	0.725	38 (52.05%)	133 (58.08%)	0.80 (0.41–1.57)	0.524
TT	17 (27.87%)	44 (18.26%)	1.65 (0.69–3.91)	0.259	18 (24.66%)	43 (18.78%)	1.20 (0.54–2.66)	0.650
CT + TT	48 (78.69%)	184 (76.35%)	1.08 (0.53–2.18)	0.839	56 (76.71%)	176 (76.86%)	0.94 (0.50–1.76)	0.839

For those polymorphisms with few homozygous variant alleles, only the combined results of the heterozygous and homozygous variant alleles are shown.

^*^Adjusted for age, gender, smoking and hypertension in multivariate unconditional logistic regression model. The P values < 0.05 are indicated in bold.

**Table 4:** Polymerase chain reaction primers and amplicons.

SNP	Alleles	Primers for PCR amplification	Amplicon (bp)	Annealing Temperatures (°C)
TaqI (rs731236)	T/C	F: 5′-CCTGTGCCTTCTTCTCTAT	171	51.9
		R: 5′-CTAGCTTCTGGATCATCTTG		52.6
BsmI (rs1544410)	G/A	F: 5′- ATATAGGCAGAACCATCTCT	166	50.5
		R: 5′-TCTGAGGAACTAGATAAGCA		50.2
Cdx-2 (rs11568820)	A/G	F: 5′-AGGGAGGAAGGAAGGAAA	193	56.2
		R: 5′-CTGTAGCAATGAAAGCAAAC		53.7
ApaI (rs7975232)	C/A	F: 5′-GAGAAGAAGGCACAGGAG	118	53.2
		R: 5′-CGGTCAGCAGTCATAGAG		52.4
FokI (rs2228570)	C/T	F: 5′-CACTGACTCTGGCTCTGA	173	52.8
		R: 5′-CTTCACAGGTCATAGCATTG		54.2
