# Supplementary material for: The vitamin D receptor gene ApaI polymorphism is associated with increased risk of renal cell carcinoma in Chinese population
Source: Sci Rep. 2016 May 13;6:25987. doi: 10.1038/srep25987 (PMC4865864; doi:10.1038/srep25987)
Supplement: Supplementary Information [file srep25987-s1.doc]

**The vitamin D receptor gene ApaI polymorphism is associated with increased risk of renal cell carcinoma in Chinese population**

Chunming Yang^1^. Jia LI^2^. Yan Li^3^. Di Wu^3^. Chengguang Sui^3^. Youhong Jiang^3^. Fandong Meng^3,*^

^1^ Department of Urology, the First Hospital of China Medical University, Shenyang 110001, China

^2^ Blood Collecting Centers, the First Hospital of China Medical University, Shenyang 110001, China

^3^ Molecular Oncology Department of Cancer Research Institution, the First Hospital of China Medical University, Shenyang 110001, China

* Corresponding author: Fandong Meng

Molecular Oncology Department of Cancer Research Institution, the First Hospital of China Medical University, Shenyang 110001, China

E-mail address: dd528@126.com

Tel: +86 13804973701

Table S1. ORs and 95%CI for all RCC cases and controls by genotypes in the VDR according to smoking status

| Genotype |  | Smoking, n (%) | | |  | OR (95% CI)* |  | *P* value* |  | No smoking, n (%) | | |  | OR (95% CI)* |  | *P* value* |
| --- | --- | --- | --- | --- | --- | --- | --- | --- | --- | --- | --- | --- | --- | --- | --- | --- |
|  |  | Case(n=98) |  | Control(n=68) |  |  |  |  |  | Case(n=204) |  | Control(n=234) |  |  |  |  |
| TaqI (rs731236) |  |  |  |  |  |  |  |  |  |  |  |  |  |  |  |  |
| TT |  | 82 (83.67%) |  | 62 (91.18%) |  | 1.00 (reference) |  |  |  | 179 (87.75%) |  | 210 (89.74%) |  | 1.00 (reference) |  |  |
| CT+CC |  | 16 (16.33%) |  | 6 (8.82%) |  | 2.27 (0.81-6.33) |  | 0.118 |  | 25 (12.25%) |  | 24 (10.26%) |  | 1.25 (0.69-2.28) |  | 0.460 |
| BsmI (rs1544410) |  |  |  |  |  |  |  |  |  |  |  |  |  |  |  |  |
| GG |  | 81 (82.65%) |  | 61 (89.71%) |  | 1.00 (reference) |  |  |  | 174 (85.29%) |  | 204 (87.18%) |  | 1.00 (reference) |  |  |
| AG+AA |  | 17 (17.35%) |  | 7 (10.29%) |  | 2.04 (0.77-5.43) |  | 0.153 |  | 30 (14.71%) |  | 30 (12.82%) |  | 1.18 (0.68-2.04) |  | 0.553 |
| Cdx-2 (rs11568820) |  |  |  |  |  |  |  |  |  |  |  |  |  |  |  |  |
| AA |  | 33 (33.67%) |  | 18 (26.47%) |  | 1.00 (reference) |  |  |  | 67 (32.84%) |  | 80 (34.19%) |  | 1.00 (reference) |  |  |
| AG |  | 51 (52.04%) |  | 39 (57.35%) |  | 0.64 (0.31-1.35) |  | 0.244 |  | 102 (50.00%) |  | 112 (47.86%) |  | 1.12 (0.73-1.72) |  | 0.592 |
| GG |  | 14 (14.29%) |  | 11 (16.18%) |  | 0.76 (0.28-2.11) |  | 0.602 |  | 35 (17.16%) |  | 42 (17.95%) |  | 1.01 (0.58-1.76) |  | 0.981 |
| ApaI (rs7975232) |  |  |  |  |  |  |  |  |  |  |  |  |  |  |  |  |
| CC |  | 39 (39.80%) |  | 37 (54.41%) |  | 1.00 (reference) |  |  |  | 75 (36.76%) |  | 112 (47.86%) |  | 1.00 (reference) |  |  |
| CA |  | 47 (47.96%) |  | 30 (44.12%) |  | 1.87 (0.94-3.72) |  | 0.073 |  | 106 (51.96%) |  | 105 (44.87%) |  | **1.51 (1.01-2.25)** |  | **0.044** |
| AA |  | 12 (12.24%) |  | 1 (1.47%) |  | **12.8 (1.52-108.55)** |  | **0.019** |  | 23 (11.27%) |  | 17 (7.26%) |  | **2.06 (1.03-4.13)** |  | **0.042** |
| FokI (rs2228570) |  |  |  |  |  |  |  |  |  |  |  |  |  |  |  |  |
| CC |  | 20 (20.41%) |  | 16 (23.53%) |  | 1.00 (reference) |  |  |  | 50 (24.51%) |  | 63 (26.92%) |  | 1.00 (reference) |  |  |
| CT |  | 59 (60.20%) |  | 42 (61.76%) |  | 0.99 (0.45-2.20) |  | 0.984 |  | 112 (54.90%) |  | 117 (50.00%) |  | 1.21 (0.76-1.91) |  | 0.419 |
| TT |  | 19 (19.39%) |  | 10 (14.71%) |  | 1.25 (0.44-3.59) |  | 0.674 |  | 42 (20.59%) |  | 54 (23.08%) |  | 0.94 (0.57-1.70) |  | 0.944 |

For those polymorphisms with few homozygous variant alleles, only the combined results of the heterozygous and homozygous variant alleles are shown.

*Adjusted for age, gender and hypertension in multivariate unconditional logistic regression model. The P values <0.05 are indicated in bold.

Table S2. ORs and 95%CI for all RCC cases and controls by genotypes in the VDR according to hypertension status

| Genotype |  | Hypertension, n (%) | | |  | OR (95% CI)* |  | *P* value* |  | No hypertension, n (%) | | |  | OR (95% CI)* |  | *P* value* |
| --- | --- | --- | --- | --- | --- | --- | --- | --- | --- | --- | --- | --- | --- | --- | --- | --- |
|  |  | Case(n=85) |  | Control(n=62) |  |  |  |  |  | Case(n=217) |  | Control(n=240) |  |  |  |  |
| TaqI (rs731236) |  |  |  |  |  |  |  |  |  |  |  |  |  |  |  |  |
| TT |  | 70 (82.35%) |  | 56 (90.32%) |  | 1.00 (reference) |  |  |  | 191 (88.02%) |  | 216 (90.00%) |  | 1.00 (reference) |  |  |
| CT+CC |  | 15 (17.65%) |  | 6 (9.68%) |  | 2.34 (0.82-6.69) |  | 0.112 |  | 26 (11.98%) |  | 24 (10.00%) |  | 1.25 (0.69-2.26) |  | 0.456 |
| BsmI (rs1544410) |  |  |  |  |  |  |  |  |  |  |  |  |  |  |  |  |
| GG |  | 70 (82.35%) |  | 55 (88.71%) |  | 1.00 (reference) |  |  |  | 185 (85.25%) |  | 210 (87.50%) |  | 1.00 (reference) |  |  |
| AG+AA |  | 15 (17.65%) |  | 7 (11.29%) |  | 2.03 (0.74-5.58) |  | 0.168 |  | 32 (14.75%) |  | 30 (12.50%) |  | 1.22 (0.71-2.09) |  | 0.470 |
| Cdx-2 (rs11568820) |  |  |  |  |  |  |  |  |  |  |  |  |  |  |  |  |
| AA |  | 30 (35.29%) |  | 17 (27.42%) |  | 1.00 (reference) |  |  |  | 70 (32.26%) |  | 81 (33.75%) |  | 1.00 (reference) |  |  |
| AG |  | 43 (50.59%) |  | 35 (56.45%) |  | 0.62 (0.28-1.34) |  | 0.222 |  | 110 (50.69%) |  | 116 (48.33%) |  | 1.13 (0.75-1.71) |  | 0.567 |
| GG |  | 12 (14.12%) |  | 10 (16.13%) |  | 0.76 (0.26-2.22) |  | 0.615 |  | 37 (17.05%) |  | 43 (17.92%) |  | 1.03 (0.58-1.74) |  | 0.941 |
| ApaI (rs7975232) |  |  |  |  |  |  |  |  |  |  |  |  |  |  |  |  |
| CC |  | 32 (37.65%) |  | 33 (53.22%) |  | 1.00 (reference) |  |  |  | 82 (37.79%) |  | 115 (47.92%) |  | 1.00 (reference) |  |  |
| CA |  | 43 (50.59%) |  | 27 (43.55%) |  | 2.09 (0.99-4.36) |  | 0.051 |  | 110 (50.69%) |  | 108 (45.00%) |  | 1.43 (0.97-2.10) |  | 0.075 |
| AA |  | 10 (11.76%) |  | 2 (3.22%) |  | **6.19 (1.18-32.46)** |  | **0.031** |  | 25 (11.52%) |  | 17 (7.08%) |  | **2.10 (1.06-4.15)** |  | **0.033** |
| FokI (rs2228570) |  |  |  |  |  |  |  |  |  |  |  |  |  |  |  |  |
| CC |  | 18 (21.18%) |  | 15 (24.19%) |  | 1.00 (reference) |  |  |  | 52 (23.96%) |  | 64 (26.67%) |  | 1.00 (reference) |  |  |
| CT |  | 52 (61.18%) |  | 38 (61.29%) |  | 1.11 (0.36-3.43) |  | 0.851 |  | 119 (54.84%) |  | 121 (50.42%) |  | 1.21 (0.77-1.90) |  | 0.398 |
| TT |  | 15 (17.65%) |  | 9 (14.52%) |  | 1.04 (0.45-2.38) |  | 0.935 |  | 46(21.20%) |  | 55 (22.92%) |  | 1.03 (0.60-1.77) |  | 0.912 |

For those polymorphisms with few homozygous variant alleles, only the combined results of the heterozygous and homozygous variant alleles are shown.

*Adjusted for age, gender and smoking in multivariate unconditional logistic regression model. The P values <0.05 are indicated in bold.
